# African Arowana Genome Provides Insights on Ancient Teleost Evolution

**DOI:** 10.1016/j.isci.2020.101662

**Published:** 2020-10-09

**Authors:** Shijie Hao, Kai Han, Lingfeng Meng, Xiaoyun Huang, Wei Cao, Chengcheng Shi, Mengqi Zhang, Yilin Wang, Qun Liu, Yaolei Zhang, Haixi Sun, Inge Seim, Xun Xu, Xin Liu, Guangyi Fan

**Affiliations:** 1 BGI Education Center, University of Chinese Academic of Sciences, Shenzhen 518083, China; 2 BGI-Qingqao, BGI-Shenzhen, Qingdao, 266555, China; 3 BGI-Shenzhen, Shenzhen 518083, China; 4 State Key Laboratory of Agricultural Genomics, BGI-Shenzhen, Shenzhen 518083, China; 5 Department of Biotechnology and Biomedicine, Technical University of Denmark, Lyngby, 2800, Denmark; 6 Integrative Biology Laboratory, College of Life Sciences, Nanjing Normal University, Nanjing, 210046, China; 7 School of Biology and Environmental Science, Queensland University of Technology, Brisbane 4102, QLD, Australia; 8 Guangdong Provincial Key Laboratory of Genome Read and Write, BGI-Shenzhen, Shenzhen 518120, China

**Keywords:** Evolutionary Biology, Phylogenetics, Paleobiology, Paleogenetics

## Abstract

*Osteoglossiformes* is a basal clade of teleost, evolving since the Jurassic period. The genomes of *Osteoglossiformes* species would shed light on the evolution and adaptation of teleost. Here, we established a chromosome-level genome of African arowana. Together with the genomes of pirarucu and Asian arowana, we found that they diverged at ∼106.1 million years ago (MYA) and ∼59.2 MYA, respectively, which are coincident with continental separation. Interestingly, we identified a dynamic genome evolution characterized by a fast evolutionary rate and a high pseudogenization rate in African arowana and pirarucu. Additionally, more transposable elements were found in Asian arowana which confer more gene duplications. Moreover, we found the contraction of olfactory receptor and the expansion of UGT in African arowana might be related to its transformation from carnivore to be omnivore. Taken together, we provided valuable genomic resource of *Osteoglossidae* and revealed the correlation of biogeography and teleost evolution.

## Highlights

• An evolutionary model of *Osteoglossidae* along the continental drift is provided
• A faster evolving rate of African arowana than Asian arowana is revealed
• The gene duplications of Asian arowana are related to more class I TE insertions
• A mechanism of African arowana’s feeding habits transition is proposed.

## Graphical Abstract

**Figure:**
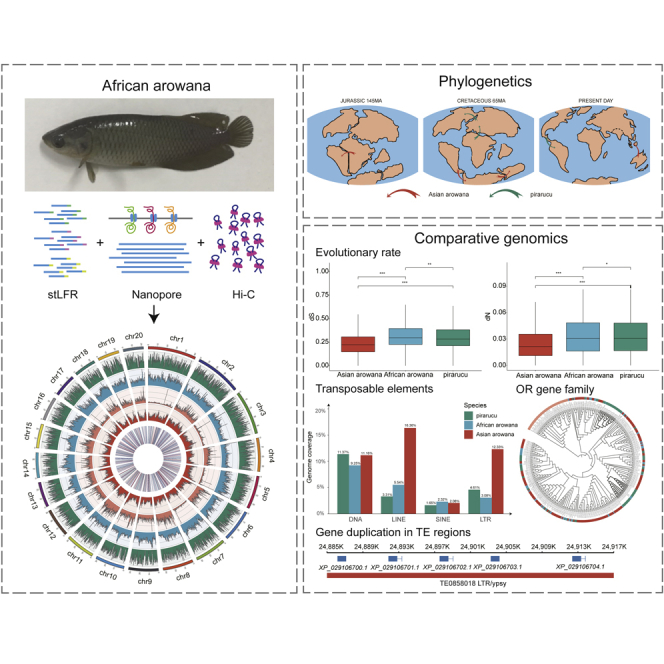

## Introduction

*Osteoglossiformes* is an ancient group of teleosts, which comprises five living groups including *Hiodontidae*, *Osteoglossidae*, *Pantodontidae*, *Notopteridae* and *Mormyridae*. *Osteoglossidae* contains two clades of *Osteoglossinae* and *Heterotidinae*, with species distributing in Asia, America, Africa, and Australia (Wilson and Murray, 2008). The existence of *Osteoglossiformes* can be dated back to the Jurassic period according to fossil evidences (Lavoue and Sullivan, 2004; Wilson and Murray, 2008), thus current species in *Osteoglossiformes* should had witnessed the break-up of the Gondwana supercontinent (Cioffi et al., 2019; Kumazawa and Nishida, 2000; Lavoue, 2016). Therefore, *Osteoglossiformes* species, serving as models for biogeography, have been extensively studied in morphological and molecular evolution (Hilton, 2001, 2003; Kumazawa and Nishida, 2000; Lavoue and Sullivan, 2004; TANG et al., 2004), and also provide evidences for paleogeology. Previous efforts have been made to decode genomes of *Osteoglossiformes* species (Bian et al., 2016; Du et al., 2019; Gallant et al., 2017), while more whole-genome sequences, especially those with chromosome information and comprehensive genome comparisons, which would further illustrate the evolutionary process of *Osteoglossiformes*.

African arowana (or African bonytongue, *Heterotis niloticus*), pirarucu (*Arapaima gigas*), and Asian arowana (*Scleropages formosus*) are three representative species of *Osteoglossidae* in *Osteoglossiformes* with some morphological differences (Adite et al., 2017; Axelrod et al., 1986; saint-Paul, 1986). African arowana is the only omnivore in *Osteoglossiformes* (Adite et al., 2013; Oliveira et al., 2019), distributing majorly in Africa, compared to pirarucu mainly in South America and Asian arowana in Southeast Asia. Despite their differences in habitats and morphology, these three species are relatively closely related with similar behaviors and physiological characters (Monentcham et al., 2009; Núñez et al., 2011; Scott and Fuller, 1976), making them good representative species for investigating the genetic basis of the ancient teleost clade (Betancur et al., 2017). In this study, we assembled the genome of African arowana using advanced sequencing and library-building technologies. In addition, together with the available genome sequences of Asian arowana and pirarucu, we comprehensively analyzed the genome evolution of *Osteoglossiformes* and illustrated the evolutionary features of *Osteoglossiformes*.

## Results

### Sequencing and Assembly of a Chromosome-Level African Arowana Genome

**Figure 1:**
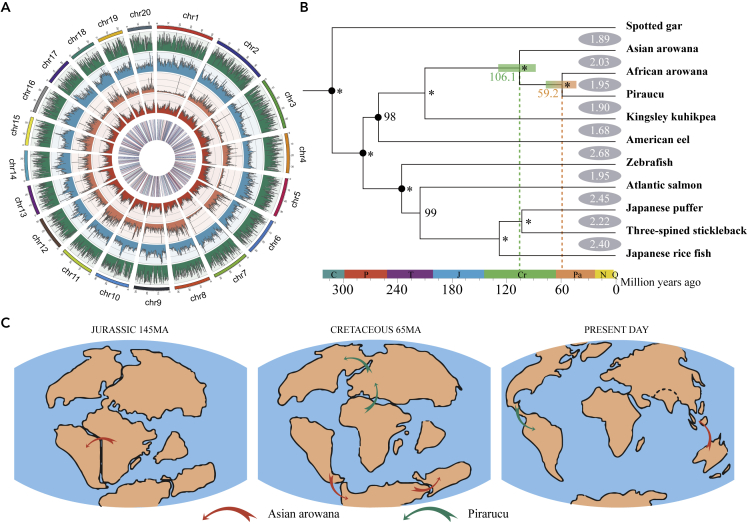
The Evolution History of Asian arowana, African arowana and Pirarucu (A) The characteristics of the assembled *H. niloticus* genome. The tracks from outer to inner represent the gene density, TE density, tandem repeat density, GC content and non-coding RNA respectively. (B) The phylogenetic relationships of 10 teleost fishes with *L. oculatus* as outgroup. The numbers on clades represent the evolutionary rates (dN + dS). The numbers beside the inner nodes represent the support values of the nodes (The asterisk represents a support value of 100). (C) pirarucu and Asian arowana’ divergence pattern through the continents drift.

In order to sequence and assemble the African arowana genome, we applied single tube long fragment read (stLFR) technology (Wang et al., 2018) on BGISEQ-500 sequencing platform and generated 144.36 Gb (∼186×) data (Table S1). In total, ∼669.7 Mb (∼99% of the estimated genome size, 673.41Mb, Figure S1) genomic sequences were assembled with a scaffold N50 of ∼9.62 Mb. To further improve the continuity, we sequenced ∼10.2 Gb (∼13.1×) Nanopore long reads to fill the gaps. With these long reads, the contig N50 was further improved from 255.6 Kb to 2.31 Mb (Table S2) using TGS-GapCloser (Xu et al., 2019). To anchor the scaffold sequences to chromosomes, we constructed a Hi-C library and sequenced ∼21.2 Gb Hi-C data and thus ∼650.44 Mb sequences were anchored to 20 chromosomes (Figures 1A, S2, and Table S3), which was consistent with the previous report on African arowana karyotype (Oliveira et al., 2019). The complete African arowana mitochondria genome was assembled using MitoZ (Meng et al., 2019), and it was almost identical to the published African arowana mitochondrial genome (Figure S3), thus indicating the correctness of sampling and species identification. Finally, by using BUSCO (Benchmarking Universal Single-Copy Orthologs) (Simao et al., 2015), we found that ∼97.6% of the complete vertebrate BUSCO genes were covered by our assembly(Table S4), providing further evidence for the fine quality of the assembled genome.

We then carried out genome annotation to identify repeats and protein-coding genes of African arowana. About 18.74% of this genome was annotated and identified as “repetitive sequences”, and DNA transposable elements (TEs) are the most abundant. We also predicted 24,146 protein-coding genes with combinational annotation methods (*de novo* prediction, homology-based prediction and RNA-seq-based prediction) in this genome (Table S3), of which ∼89.5% were found to have homologs in public databases (Kyoto Encyclopedia of Genes and Genomes, Swiss-Prot, Translated EMBL, NCBI non-redundant proteins) with known functions (Table S5). Clustering with 10 other fishes, we identified 15,432 gene families in African arowana, of which 30 are unique to African arowana.

### Speciation of *Osteoglossiformes* along with the Geographic Drift

To validate and add to the previously proposed model of Gondwana origin and plate tectonic transportation of *Osteoglossiformes* species (Kumazawa and Nishida, 2000), we constructed the phylogenetic tree of the representative species in *Osteoglossiformes*. Collecting the ten teleost species (African arowana, Asian arowana, pirarucu, American eel (*Anguilla rostrate*), Atlantic salmon (*Salmo salar*), Kingsley kuhikpea (*Paramormyrops kingsleyae*), Japanese puffer (*Takifugu rubripes*), Japanese rice fish (*Oryzias latipes*), three-spined stickleback (*Gasterosteus aculeatus*), Zebrafish (*Danio rerio*)) with available genome sequences and spotted gar (*Lepisosteus oculatus*) as an outgroup species, we identified 355 single-copy gene families (one orthologous gene in each species) and then used them to build the phylogenetic tree, reflecting the relationship and evolution of teleost (Figure 1B). The divergence time of each internal node was calculated using MCMCTree (Yang, 2007) with the calibration of previous molecular or fossil researches obtained from TimeTree (http://www.timetree.org/, Table S6). In this phylogenetic tree, the divergence time between Asian arowana and the common ancestor of African arowana and pirarucu was ∼106.1 million years ago (MYA), which was moderate to previous researches (Cioffi et al., 2019; Du et al., 2019; Vialle et al., 2018). And the divergence time estimated here was close to the final separation time of South American and African continents in Afro-South American drift of Gondwana supercontinent happened at ∼110 MYA (Rogers and Santosh, 2004). Considering the previous evidences supporting that (1) *Osteoglossinae* fishes speciated along with the separation of South America, Antarctic, Australia, and Southeast Asia from ∼50.3MYA (Cioffi et al., 2019); (2) Presently, Asian arowana only lives in Southeast Asia (Mu et al., 2012); and (3) Africa has been identified as the taxonomic diversity center of *Osteoglossiformes* (Wilson and Murray, 2008), we proposed that the ancestor of Asian arowana had migrated from Africa to Southeast Asia before or during the tectonic-mediated Gondwanan fragmentation (especially the fragmentation of Africa-South America, South America-Antarctica-Australia and the fragmentation of Southeast Asia-Australia) (Figure 1C). Then, the divergence time between African arowana and pirarucu was estimated as ∼59.2 MYA (Figure 1B), which was also close to the split time of North American and Eurasian continents (∼65 MYA or later) (Rafferty, 2010; Rogers and Santosh, 2004). Additionally, a Paleocene (56–65Ma) *Heterotidinae* fossil was discovered in North America (Guo-Qing and Wilson, 1996) and classified as an outgroup of African arowana and pirarucu (Wilson and Murray, 2008). Thus, we concluded that the ancestor of African arowana and pirarucu might live in both North America and Eurasia continents of Gondwana supercontinent, and after the split of these continents, the two species have evolved separately, while pirarucu had spread to South America after the formation of Isthmus of Panama (Figure 1C). Hence, by using the whole-genome data analysis, we proposed the association between the speciation of *Osteoglossiformes* species and the paleo-geographical changes and improved the previous model for speciation of these species.

### Main Distinct Genomic Evolution Events of Three *Osteoglossidae* Fishes during Their Adaptations to New Environments

#### Dynamic Evolution Rate

**Figure 2:**
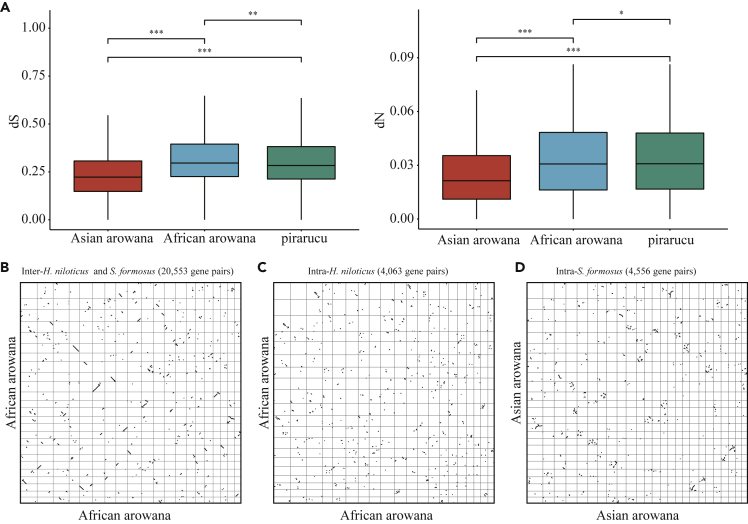
The Evolution Rate of Asian arowana, African arowana and Pirarucu (A) The dS and dN distribution of Asian arowana, African arowana, and pirarucu. The statistic significancy was calculated by Wilcoxon rank-sum test and three asterisks indicate a p value that less than 2.22∗e−16 and two asterisks indicate a p value that equal to 2.5 × 10^−11^ while one asterisks indicate a p value that equal to 0.9. (B) Syntenic pattern of African arowana and Asian arowana. (C) Syntenic pattern of intra-African arowana. (D) Syntenic pattern of intra-Asian arowana.

In addition to phylogenetic analysis, we further investigated these three genomes in detail to reveal the genome evolution during the long period after speciation. First, we calculated the dN and dS (the substitution rates at non-synonymous and synonymous sites) of 355 single-copy gene families in each clade. Comparing to the common ancestor, we found that the dN and dS values of Asian arowana (average dN: 0.024; average dS: 0.253) were lower than those of African arowana (average dN: 0.032; average dS: 0.317) and pirarucu (average dN: 0.032; average dS 0.307), indicating higher mutation rate and faster evolution in African arowana and pirarucu compared to Asian arowana (Figure 1B). In order to further validate these results, we identified 7,699 single-copy gene families among three *Osteoglossidae* fishes and spotted gar. For these orthologous gene families, we calculated the average dN and dS of these three *Osteoglossidae* species and also found that the dN and dS of African arowana (average dN: 0.039; average dS: 0.335) and pirarucu (average dN: 0.040; average dS: 0.346) were significantly greater than those of Asian arowana (average dN: 0.027; average dS: 0.242). The Wilcoxon rank-sum test showed that the differences between Asian arowana and either African arowana or pirarucu were statistically significant (p-value<0.05, Figure 2A). Besides, we investigated the number of pseudogenes (the remains of malfunctional genes because of mutation accumulation) of these three *Osteoglossidae* species and found more pseudogenes in African arowana (350) and pirarucu (399) than in Asian arowana (242).

We also explored the evolution rate of these three *Osteoglossidae* species' conserved regions. Whole-genome alignments of these three *Osteoglossidae* species were implemented firstly with spotted gar as a reference. Then, we calculated the genetic distance (TN93 model) in the resulting 30 Mb conserved regions (remain in four species) between each *Osteoglossidae* species and spotted gar, respectively. And this analysis revealed that the average distance of African arowana (0.358) was significantly larger than Asian arowana (0.353) and pirarucu (0.351) (Wilcoxon rank-sum test, p value<2.22 × 10^−16^, Figure S4). Moreover, we identified syntenic blocks of African arowana and Asian arowana to further investigate the evolution of their genomes. We found a distinct colinear relationship between African arowana and Asian arowana, indicating slight chromosomal structural variations occurred between their genomes (Figure 2B). We also found a rough chromosomal one-to-one colinear relationship in Asian arowana itself, whereas more scattered self-syntenic blocks were detected in African arowana genome than in Asian arowana (Figures 2C and 2D). These results provided additional evidences supporting the faster evolution rate of African arowana, which had resulted in more variations in the paralogous chromosomes inheriting from the TS-WGD events (∼350 MYA) (Glasauer and Neuhauss, 2014).

#### Extra Class I TE Insertion in Asian Arowana

**Figure 3:**
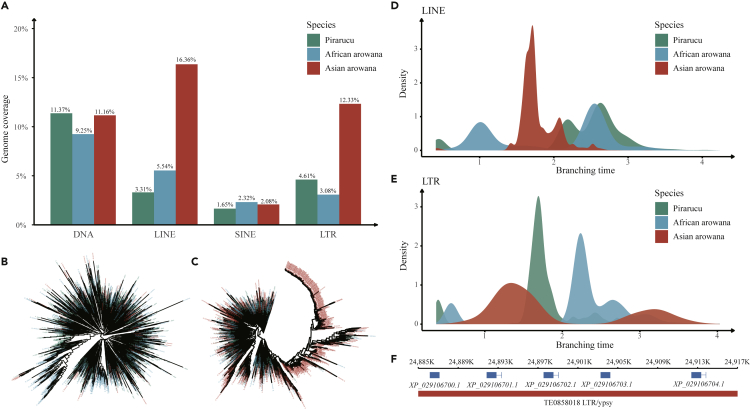
Transposable Element (TE) Dynamics of Pirarucu, African Arowana and Asian Arowana (A) The distribution of three *Osteoglossidae* fishes' TEs. (B) The phylogenetic trees of three *Osteoglossidae* fishes' Tc1 TEs. (C) The phylogenetic trees of three *Osteoglossidae* fishes' Tigger TEs. (D) The insertion timeline of LINE TEs of three *Osteoglossidae* fishes. (E) The insertion timeline of LTR TEs of three *Osteoglossidae* fishes. (F) An example of the positional relationship of Asian arowana's genes and TEs, in which the blue bars indicate the genes and the red bar indicate the TEs. In this case, all of the five genes are olfactory receptors.

Other than the faster evolution rate in African arowana and pirarucu genomes, the genome sizes of these two species (669 Mb and 667 Mb, respectively) are also notably smaller than that of the Asian arowana genome (785 Mb). Thus, we then investigated the possible mechanisms underlying the smaller genome sizes. Performing the same combinational annotation methods (*de novo-* and homology-based methods), we annotated the TEs in all three *Osteoglossidae* fishes. Looking into the repeat content, we found it was substantially less in African arowana and pirarucu genomes (18.7% and 18.2%, respectively) than in Asian arowana genome (29.5%). The extra insertion in repeat content (∼100 Mb) may explain the differences of genome sizes (Table S7). Further investigating the different categories of TEs, we found the LINEs (long interspersed nucleotide elements) and LTRs (long terminal repeats) proportions were notably different among African arowana (LINEs: 5.54%, 37.10 Mb and LTRs: 3.08%, 20.63 Mb), pirarucu (LINEs: 3.31%, 22.11 Mb and LTRs: 4.61%, 30.74 Mb) and Asian arowana (LINEs: 16.36%, 128.34 Mb and LTRs: 12.33%, 96.77 Mb) (Figure 3A). Although, DNA TE proportions were comparable in 3 species, the most abundant DNA TE class of Asian arowana was TcMar-Tigger while TcMar-Tc1 was the most abundant in both African arowana and pirarucu, and the distinctly expanded clade of TcMar-Tigger of Asian arowana indicated a recent fast insertion event (Figures 3B and 3C).

Additionally, we estimated the relative insertion time of LINEs and LTRs through the Ty3 reverse transcriptase (RT) genetic distance to the outgroup (all the Ty1 LTR RTs sequences of Asian arowana) and found that Asian arowana had a different LINE insertion time peak compared to African arowana and pirarucu, as well as an extra LTR insertion time peak which was close to its LINE insertion peak, indicating that Asian arowana had experienced a specific period (Figures 3D and 3E). To further figure out the function of the additional TEs insertion event, we investigated the TE coverage of all genes of these 3 species. In Asian arowana, we identified 1,261 genes located in TE-inserted regions, while this number in pirarucu and African arowana was only 464 and 73, respectively. The genes covered by TEs were concatenated together and had the same function (Figures 3F and S5). Functional enrichment analysis showed that these genes in Asian arowana were mainly related to olfactory transduction, NOD-like receptor signaling and phagosome pathways (Table S8, q-value<0.01). In summary, we found the genome of Asian arowana had gone through more changes due to the extra insertion of TEs, and also multiple gene families had expanded along with the copy and paste of TEs, which might be related to their adaptions to more variable environment after the first Gondwana split event than African arowana and pirarucu.

#### Dynamic Evolution of Gene Families

**Figure 4:**
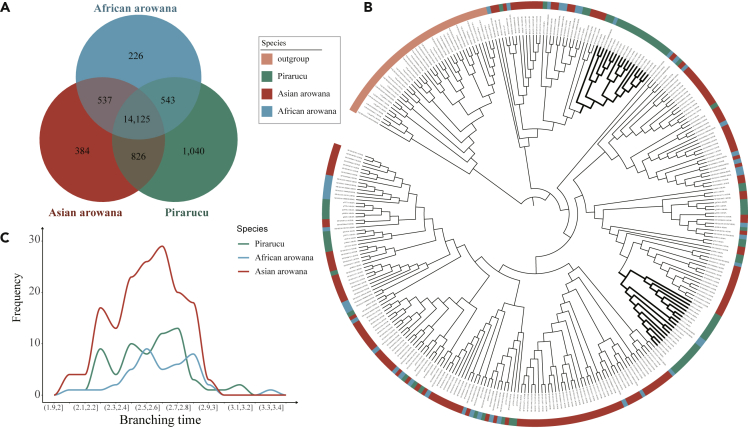
Gene Family Dynamics of Three *Osteoglossidae* Fishes (A) The venn diagram represents the overlap relationship of three *Osteoglossidae* fishes' gene families. (B) The gene tree of three *Osteoglossidae* fishes' *OR* genes in which the bold clades indicated two gene expansions of pirarucu. (C) The insertion timeline of *OR* genes.

Since we have observed a higher evolution rate in African arowana and more TEs insertion in Asian arowana, their effects on gene content were further evaluated by gene family analysis. We found the average gene family size of African arowana (1.38 gene per family) was less than Asian arowana and pirarucu (both 1.47 gene per family), possibly indicating a higher evolutionary rate based on a published research (Chen et al., 2010). Overall, 14,125 gene families were shared by all these three species, and 1,040 gene families were unique to pirarucu (Figure 4A) which was more than that in Asian arowana (384) and African arowana (226). The unique gene families of Asian arowana were related to salivary secretion and olfactory transduction, whereas those of pirarucu were related to cell growth and death (necroptosis and apoptosis), and cellular community (tight junction, adherens junction, gap junction, and focal adhesion). Additionally, eight *UGT* (*K00699*) genes were found in the unique gene families of African arowana. *UGT* genes have been shown to play a critical role in many metabolism pathways such as ascorbate and aldarate metabolism, retinol metabolism, steroid hormone biosynthesis, and porphyrin & chlorophyll metabolism, which might be related to its special omnivorous diet character (Kakehi et al., 2015; Kawai et al., 2018) (Table S9, q-value<0.01).

We then detected 1,210, 424, and 829 expanded gene families in pirarucu, African arowana, and Asian arowana, respectively, (Figure S6). KEGG functional enrichment analysis of these expanded gene families showed that both pirarucu and Asian arowana had experienced significant expansion in 14 pathways including olfactory transduction, salivary secretion, cell adhesion molecules (CAMs) and NOD-like receptor signaling, which were not found in African arowana (Table S10, q-value<0.01). In contrast, the gene family contraction of African arowana (2,510) was more than Asian arowana (2,010) and pirarucu (1,151), which was related to olfactory transduction, tight junction, cellular senescence, TGF-beta signaling pathway (Table S11, q-value<0.01). More importantly, the expansion of *UGT* genes in African arowana further led to the expansion of several metabolic pathways.

### Possible Genetic Mechanisms Underpinning Diet Change of African Arowana

African arowana is an omnivore that has a wide range of preys including small benthic fishes, shrimps, plants, and insects (Adite et al., 2013; Monentcham et al., 2009). In contrast, its closely related species, pirarucu and Asian arowana, are predominantly dependent on fish preys (Natalia, 2004; Saint-Paul, 1986). In order to explore the genetic mechanisms of diet change in African arowana, we comprehensively examined the taste receptors of all tastes including sweet, umami, bitter, sour, and salty in these three genomes and found no significant differences in expansion or contraction (Table S11). The vertebrates have three kinds of odorant receptors including olfactory receptors (*ORs*), vomeronasal receptors *V1R* and *V2R* (Alioto and Ngai, 2005). Thus, then we carried out a comparative analysis of the odorant receptors of these three genomes. The *V1R* genes and *V2R* genes in pirarucu, African arowana, and Asian arowana showed no obvious expansion (Table S12). However, we found that *OR* genes (*K04257*) were significantly contracted in African arowana (40) comparing with pirarucu (70) and Asian arowana (160). Through the gene tree of *ORs*, we further observed that Asian arowana had kept more gene copies in most clades of *OR*, while pirarucu had experienced contraction in several clades except for two clades (marked by bold clades, Figure 4B). In addition, in African arowana, almost all *OR* gene clades contained fewer members than that of Asian arowana and pirarucu. Estimation of the expanding time for *OR* genes showed that Asian arowana had gained much more *OR* gene copies during the whole timeline and had been through an extra insertion period, which can also be observed in pirarucu while only ancient *OR* expanding events were inferred in African arowana (Figure 4C). Given the results that the olfactory transduction pathway gene family underwent dynamic evolution in history and the expansion of *UGT* genes, we concluded that the change of *OR* genes and *UGT* genes might play a key role in the diet transition of African arowana.

## Discussion

Continental drift leads to severe environmental change and geographical separation which would be the reason and driving force for differentiation and speciation (Chen et al., 2018; Dodd and Afzal Rafii, 2001). Phylogenetic relationships among those species which spread across different continents are of great interests for scientist from the establishment of plate tectonics (Casadevall et al., 2017; Sterrer, 1973; Wolfson, 1948). The evolutionary history of freshwater fish was also associated with the continental motion and discussed lively because of the ability to migrate across ocean for some species (Nakatani et al., 2011; Sparks and Smith, 2005). The *Osteoglossidae* species spread across all continents except Antarctica, serving as a typical subject to investigate the association between its speciation and continental drift. Here, we chose three representative *Osteoglossidae* species to investigate their divergence and revealed their genomics differences related to the continental motion.

We first assembled the genome of African arowana with advanced library-building and sequencing technologies because the genomes of Asian arowana and pirarucu were available. The stLFR technology was applied because of its cost-saving and convenience to get a better assembly without the need of multiple insertion libraries (Wang et al., 2018). The nanopore sequencing technology was applied to extend the genome continuity for its long reads. Also, we employed the power of Hi-C technology to link the scaffolds into pseudo-chromosomes. Eventually, we got the final African arowana genome with a better quality than recent researches about genome assemblies (Li et al., 2020; Xie et al., 2020). The genome sequences of African arowana provided important genetic resources for further researches.

The phylogenetic analysis in this study revealed a possible speciation track of *Osteoglossidae* species. Within 11 fishes' genome, we used conserved single-copy gene families which is reliable for phylogeny construction (Aguileta et al., 2008) to reveal the speciation history. By using MCMCtree and the calibration of published speciation time, we surprisingly found both the divergence time between African arowana & Asian arowana, and African arowana & pirarucu were consistent with the time of continental separation. The coincidences were supported by their geographical distribution and the fact that Africa is the biodiversity center of *Osteoglossiformes* and *Heterotidinae* (includes Africa arowana and pirarucu) fossil record found in North America. Together with the model of published Asian arowana's speciation history, we proposed a more complete model to reveal the speciation history of *Osteoglossidae*. Our model not only provides the possible speciation path of Asian arowana and pirarucu but will also guide the researches on paleontology in the future. Moreover, our results also hint a possibility of population genetic research to investigate their population history.

To reveal the evolutionary process they experienced, we focused on the genomic difference of three *Osteoglossidae* species. The faster evolutionary rate, less expansion and more contraction of gene families of African arowana were found. The association of evolutionary rate and dynamic changes of gene families was investigated previously, hinting a possible causality between African arowana's faster evolution rate and gene family contraction (Chen et al., 2010). We also found more class I TE insertions together with more genes covered by TE insertions which are concatenated in position and duplicated in function in Asian arowana genome. Published researches had reported the relation of genes and TEs in human genome and plant genome and had interpreted the effect of TEs on gene creation, gene evolution and genome rearrangement (Bennetzen, 2005; Nekrutenko and Li, 2001). Therefore, the evolutionary dynamics of gene families in African arowana and more TE insertions of Asian arowana probably play a key role in their adaptive process to new environments. Moreover, we identified an expansion of SINE/5S elements in pirarucu whose function need to be further characterized (Figure S7).

We observed a significant difference in gene number of *OR* family among the three *Osteoglossidae* fishes, while the taste receptors, other odorant receptors *V1R* and *V2R* were conserved among them. Together with the expansion of *UGT* family in African arowana genome, we proposed a possible genetic mechanism underlying the diet change of African arowana. Diet change and the genomic evolution process had been investigated broadly (Perry et al., 2007; Schondube et al., 2001). Our research provides a case to investigate this phenomenon and a view to explain this process. However, when this change started and whether it happened on pirarucu is unknown because pirarucu experienced a similar contraction to African arowana in several clades of *OR* genes.

### Limitations of the Study

The data provided here are not sufficient to answer all questions we put forward. More researches will be required to conduct in the future such as the fossils evidence searching in Africa and South America. The impact of TEs insertion on new gene and gene expression regulation in Asian arowana also needs further study. The diet transition of African arowana and the inter-continental emigration of bonytongues should be transferable environments of freshwater fishes particularly for those living in the same period and similar environment with *Osteoglossidae*. Therefore, more evidences should be revealed in the future. Molecular biological experiments such as RNAi, gene knockout or genetic modification to verify the genetic mechanism underpinning the diet transition are also needed. Moreover, the additional *de novo* genomic researches and comparative genomic researches on *Osteoglossidae* and other *Osteoglossiformes* fish, which will help us to understand the evolution of this ancient teleost clade such as 10000 fish genome project (Fish10K) (Fan et al., 2020). The resequencing genomic and ancient genomic studies about these three *Osteoglossiformes* fishes were required to disclose the population structure, migration history, and genomic changes in their own genome along with the time.

### Resource Availability

#### Lead Contact

Further information and requests for resources should be directed to and will be fulfilled by the Lead Contact, Guangyi Fan (fanguangyi@genomics.cn).

#### Materials Availability

There is no resulting materials generated by this study.

#### Data and Code Availability

The accession numbers for the genome sequencing data, RNA sequencing data, and genome assembly reported in this paper are CNGBdb: CNP0001313 and NCBI: PRJNA665338.

## Methods

All methods can be found in the accompanying Transparent Methods supplemental file.

## Supplemental Information

Document S1. Transparent Methods, Figures S1–S7, and Tables S1–S13
