# Supplementary material for: African Arowana Genome Provides Insights on Ancient Teleost Evolution
Source: iScience. 2020 Oct 9;23(11):101662. doi: 10.1016/j.isci.2020.101662 (PMC7586111; doi:10.1016/j.isci.2020.101662)
Supplement: Document S1. Transparent Methods, Figures S1–S7, and Tables S1–S13 [file mmc1.pdf]

iScience, Volume *23*

Supplemental Information

African Arowana Genome Provides Insights on Ancient Teleost Evolution

Shijie Hao, Kai Han, Lingfeng Meng, Xiaoyun Huang, Wei Cao, Chengcheng Shi, Mengqi Zhang, Yilin Wang, Qun Liu, Yaolei Zhang, Haixi Sun, Inge Seim, Xun Xu, Xin Liu, and Guangyi Fan

African arowana genome provides insights on ancient teleost evolution

Shijie Hao[1,2,7], Kai Han[2,7], Lingfeng Meng[1,2], Xiaoyun Huang[2], Wei Cao[3], Chengcheng Shi[1,2], Mengqi Zhang[2], Yilin Wang[2], Qun Liu[2], Yaolei Zhang[2,5], Haixi Sun[3], Inge Seim[6], Xun Xu[2,3], Xin Liu[2,3,4,*], Guangyi Fan[2,3,4,8,*].

[1]BGI Education Center, University of Chinese Academic of Sciences, Shenzhen 518083, China

[2]BGI-Qingqao, BGI-Shenzhen, Qingdao, 266555, China.

[3]BGI-Shenzhen, Shenzhen 518083, China.

[4]State Key Laboratory of Agricultural Genomics, BGI-Shenzhen, Shenzhen 518083, China.

[5]Department of Biotechnology and Biomedicine, Technical University of Denmark, Lyngby, 2800, Denmark.

[6]Comparative and Endocrine Biology Laboratory, Translational Research Institute-Institute of Health and Biomedical Innovation, School of Biomedical Sciences, Queensland University of Technology, Brisbane 4102, Queensland, Australia.

[7]These authors contributed equally.

[8]Lead Contact

[*]Correspondence: liuxin@genomics.cn (X.L.), fanguangyi@genomics.cn (G.F.).

Transparent Methods

Sample collection, library construction and genome sequencing

An individual African arowana fish from a seafood market at Xiamen, Fujian province, southeast China was collected and the muscle tissues were used for DNA extraction using the conventional salting-out method. The high molecular weight genomic DNA with an average length of 50 Kb was further used to construct a single tube Long Fragment Read (stLFR) library using the MGIEasy stLFR Library Prep kit (PN: 1000005622) according to the instructions(Wang et al., 2019). Hi-C library was constructed following the Wang's methods(Wang et al., 2019) with whole blood tissue of the same individual. The sequencing was conducted on a BGISEQ-500 platform with pair-end 100 bp read length. Besides, one nanopore library was also prepared according to the instructed protocol using the Oxford Nanopore SQK-LSK109 kit and sequenced on GridIon X5 platform.

Genome survey

The *k*-mer frequencies within the clean stLFR reads were analyzed to estimate the major genome

characteristics. The occurrences of all 17-mers within both strands were counted using jellyfish v2.2.7(Marcais and Kingsford, 2011), and the genome size, heterozygosity as well as repeat content were calculated using GenomeScope (Vurture et al., 2017). The modeling distribution of 17-mer frequency demonstrated a peak at around 52, with 41,344,762,649 total number of *k*-mers. The estimated haploid genome size was 673.41Mb, of which 31% was inferred to be repeat, a low heterozygosity rate (0.13%) was detected while the *k-mer* distribution showed no apparent peak indicated heterozygosity in this genome.

De novo genome assembly

Draft genome sequence was first assembled using Supernova v2.1.1(Weisenfeld et al., 2017) software and processed with one round of gap-closing using Gapcloser v1.12(Luo et al., 2012) with stLFR data. In this process, the stLFR reads were first pre-processed to be compatibly handled by supernova assembler, using the stLFR2Supernova pipeline (https://github.com/BGI-Qingdao/stlfr2supernova_pipeline). Then, we enhanced the draft assembly using TGS-GapCloser pipeline (Xu et al., 2019) based on the single molecular long reads.

Hi-C data were used to improve the connection integrity of the scaffolds. We first detected all valid pairs of reads using Hic-Pro v2.8.0(Servant et al., 2015) by mapping clean Hi-C reads to draft genome sequences, and the valid read pairs were extracted and aligned to the genome using Juicer v1.5(Durand et al., 2016b). Then the assembled fragments of DNA were ordered and oriented using 3D-DNA pipeline(Dudchenko et al., 2017) based on the Juicer Hi-C contacts (‘merged_nodups.txt’ file). Manual review and refinement were also performed by using Juicebox Assembly Tools v1.9.0(Durand et al., 2016a) to identify and remove the remaining assembly errors.

Genome annotation

We firstly detected and annotated the repetitive sequences in the genomes. For the annotation of TRFs, Tandem Repeats Finder v 4.04 program(Benson, 1999) was employed. The TEs were annotated by a combination of both *de novo* prediction and homology-based identification. Briefly, the genome sequences were *de novo* searched using LTR_Finder(Xu and Wang, 2007) and RepeatModeler(Smit et al., 2019) to find sequence elements with specific consensus models of putative interspersed repeats. The non-redundant self-contained repeat library was then searched against the genome using RepeatMasker (http://www.repeatmasker.org/). In the homology-based

detection, the genome sequences were aligned to both the public Repbase 21.01 and transposable element protein database (included in the RepeatMasker package) to detect interspersed repeats. Evidences including the results of ab initio gene predictors and homologous gene models to proteins previously discovered in other sequenced genomes, as well as transcript sequences were integrated to make a comprehensive gene structure annotation. The Augustus (Stanke et al., 2006), GlimmerHMM (Majoros et al., 2004) and Genescan (Burge and Karlin, 1997) were applied for ab initio gene finding with best parameters trained for zebrafish and vertebrates. For homology-based prediction, nonredundant protein sequences from 5 species (*Oreochromis niloticus*, *Pundamilia nyererei*, *Maylandia zebra*, *Astatotilapia calliptera* and *Scleropages formosus*) were aligned against African arowana genome using GeneWise v2.4.1 program(Birney et al., 2004). Furthermore, transcript sequences were constructed based on the RNA-Seq alignment to the genome that generated by using HISAT v2.1.0(Kim et al., 2019), and candidate coding regions within the transcripts were further detected, in which ORFs with homology to known proteins were also identified via blast (against SwissProt database) and pfam searches, using TransDecoder v5.5.0 (https://transdecoder.github.io/). Final consensus gene models were produced by integrating those disparate sources of gene structure evidence using GLEAN software (Elsik et al., 2007). Total 24,146 genes, covering 96.8% vertebrate benchmarking universal single-copy orthologs (BUSCOs)(Waterhouse et al., 2018), were predicted in the African arowana genome with average length 14911.23 bp. The length distributions of mRNA, coding sequences, exon and intron were closely similar to that of related species.

Functional annotations of the predicted genes were performed by aligning protein sequences using BLAST to KEGG release 84.0, Swissprot release 201709, Trembl release 201709 and Clusters of Orthologous Groups (COGs) database. The results show that 21,609 (89.49%) protein-coding genes were assigned successfully to at least one well-modeled functional category.

We also scanned matches of the protein sequences against the genomes of *Osteoglossidae* species (Aian arowana, African arowana and pirarucu) and detected the possible pseudogenes separately using PseudoPipe(Zhang et al., 2006) annotation tool. Pseudogenes overlapping a location of coding genes as well as those classified as fragment match were further discarded from the final annotation.

Evolutionary phylogeny of African arowana

To reveal the phylogenetic relationships of African arowana and other bony fishes, gene set of

five *Clupeocephala* species (*Danio rerio*, *Salmo salar*, *Oryzias latipes*, *Gasterosteus aculeatus* and *Takifugu rubripes*), one *Elopomorpha* species (*Anguilla rostrate*) and three *Osteoglossomorpha* species (*Scleropages formosus*, *Paramormyrops kingsleyae* and *Arapaima gigas*), plus one species from *Lepisosteiformes* (*Lepisosteus oculatus*) as outgroup, were downloaded from NCBI and further used to detect gene clusters. We extract the longest transcript from unique genomic loci to eliminate redundant splicing, and retained coding sequences longer than 150 bp from each dataset to discard possibly unreliable gene predictions. We performed all-versus-all BLAST search for protein sequences of these 11 species and the resultant matches were sorted out for filtering redundant and segments, then the genes were further clustered into 23,654 families using hcluster_sg tool (https://sourceforge.net/p/treesoft/code/HEAD/tree/branches/lh3/). We performed multiple sequences alignment using MUSCLE v3.7 software(Edgar, 2004) for each single-copy gene cluster and further concatenated the alignments into super-matrix. Phylogenetic relationships of these species were inferred using MrBayes v3.1.2 (Ronquist et al., 2012) based on the fourfold degenerate site of the supergene. The divergence time of our target species were also determined using MCMCTree (Yang, 2007) with the public timelines from TimeTree (Kumar et al., 2017) as calibrations. Given the phylogenetic relationship and divergence time, we analyzed the changes in gene family size using CAFE v2.1(De Bie et al., 2006). We compared the gene pairs in the paralogous and orthologous families detected by using wgd v1.0.1 package (Zwaenepoel and Van de Peer, 2019), the distribution of synonymous mutation rate (*Ks*) was used as an indicator of the duplication and divergence event in three *Osteoglossidae* species (*Scleropages formosus*, *Arapaima gigas* and *Heterotis niloticus*).

Evolutionary rate calculation

The 355 11-speceis one-to-one gene families were firstly used to calculate evolutionary rate by using the PAML v4.4c software. For every gene families, the dN and dS of non-sportted gar genes of each lineage were extracted from the result files and used for further statistics. To perform more extensive analysis, we identified one-to-one gene families among spotted gar and three *Osteoglossidae* fishes and calculated dN and dS with PAML too. Moreover, the conserved genome regions among three *Osteoglossidae* fishes and spotted gar were identified using MultiZ v1.0 software with genome alignments between every *Osteoglossidae* fish and spotted gar. Then, the genetic distances between *Osteoglossidae* fishes and spotted gar within the conserved regions were calculated using home-made python script and TN93 algorithm (**Script 1**). Finally, we

implemented syntenic analysis between Asian arowana & African arowana, Asian arowana itself and African arowana itself with Jcvi v0.8.12 toolkit.

TE insertion timeline estimation

To detect the insertion timeline of TEs within the 3 *Osteoglossidae* fish genomes, we firstly identified the transposable elements' conserved domains using DANTE pipeline (https://github.com/kavonrtep/dante). Protein sequences of RT (reverse transcriptase) domains from the most abundant TE types (here LINEs and LTR/Ty3s) were extracted for each of *Osteoglossidae* species. The sequences were further aligned, together with the same type of sequences from Asian arowana's LTR/Ty1 TEs, using MAFFT v7.453 (Katoh and Standley, 2013) program for each species. Then TE element trees were built based on the multiple sequences alignment using FastTree v2.1.10 (Price et al., 2010) and re-rooted on LTR/Ty1s to trace the evolutionary trajectory of LINEs or LTR/Ty3s. Concentration of branch length between terminal leaf and root node was calculated with a home-made python script and used to imply the explosion of TE element in each species.

Genes and transposable elements phylogenetic trees construction

We also investigated the evolutionary pathways of genes with similar function and repeat elements with the same class, within three *Osteoglossidae* fishes, based on the annotation results of RepeatMasker and KEGG (see Method). In this process, we used MAFFT program and FastTree software to perform the sequences alignment and tree building. All evolutionary trees were visualized using ggtree(Yu et al., 2017) package under R environment as well as the interactive online tool iTOL(Letunic and Bork, 2019). To screen and qualify gene trees with species-specific expanded clade, we traversed all nodes in a tree, by using an in-house python script (**script 2**) and ete3(Huerta-Cepas et al., 2016) module, to find out clade containing more than ten genes of which 80% were from the same species.

In-house Scripts

Script 1. Calculate the genetic distance of conserved genome regions.

```
#!/usr/bin/env python3

import os
import sys
```

```
from tn93 import tn93

class SEQ:
    def __init__(self, line):
        lst = line.split()
        self.source = lst[1]
        #self.name = source[0]
        #self.seqid = '.'.join(source[1:])
        self.start = int(lst[2]) + 1
        self.length = int(lst[3])
        self.strand = lst[4]
        source_length = int(lst[5])
        if self.strand == '-':
            self.end = source_length - self.start
            self.start = self.end - self.length + 1
        else:
            self.end = self.start + self.length - 1
        self.seq = lst[6].upper()

def calculate(sequences, score):
    target = sequences[0]
    length = len(target.seq)
    line = '{}:{}-{}{}'.format(target.source, target.start,
            target.end, target.strand)
    line += '\t{}\t{}'.format(length, score)
    for index, seq in enumerate(sequences[1:]):
        dist = tn93(target.seq, seq.seq, length,
                matchMode=3, min_overlap=length // 4)
        query = '{}:{}-{}{}:{:5f}'.format(seq.source, seq.start,
                seq.end, seq.strand, dist)
        line += ('\t' + query)
    return line + '\n'

if __name__ == '__main__':

    sys.stdout.write('#target\talign_length\tscore\tquery:dist\n')
    sequences = []
    r = open(sys.argv[1])
    for line in r:
        if line.startswith('#'):
            continue
        if line.startswith('a score='):
            score = line.strip().split('=')[1]
        if line.startswith('s '):
            sequences.append(SEQ(line))
        if line.startswith('\n'):
            info = calculate(sequences, score)
            sys.stdout.write(info)
```

```
            sequences = []
    r.close()
```

Scripts 2. Select expanded gene trees.

```
#!/usr/bin/env python3

import os
os.environ['QT_QPA_PLATFORM'] = 'offscreen'
import sys
import glob
from ete3 import Tree, TreeStyle, NodeStyle
indir, outdir, threshold = sys.argv[1:]

def standard(leaf_names):
    if len(leaf_names) < 10:
        return False
    else:
        FZ_names = [i for i in leaf_names if i.startswith('NH_')]
        YZ_names = [i for i in leaf_names if i.startswith('XP_')]
        if any((len(x)/len(leaf_names) >= float(threshold)) for x in [FZ_names, YZ_names]):
            return True
        else:
            return False


tree_dirs = glob.glob(os.path.join(indir, 'K*'))
dirname = lambda x:os.path.basename(x)

tree_files = [[dirname(x), os.path.join(x, '{}.pep.tree'.format(dirname(x)))]
        for x in tree_dirs]

pass_trees = []
for geneID, tree_file in tree_files:
    try:
        tree = Tree(tree_file, format=0)
    except:
        sys.stderr.write('*** Parse Error: {}\n'.format(tree_file))
        continue
    tree.convert_to_ultrametric(strategy='cladogram')
    tree.ladderize()
    tag = False
    for node in tree.traverse('levelorder'):
        leaf_names = node.get_leaf_names()
        if not standard(leaf_names):
            continue
        tag = True
    if tag:
```

```
        pass_trees.append([geneID, tree])

if not os.path.exists(outdir):
    os.makedirs(outdir)

tree_style = TreeStyle()
tree_style.mode = 'c'
tree_style.scale = 120
tree_style.show_leaf_name = False
#tree_style.optimal_scale_level = 'full'
FZnode_style = NodeStyle()
FZnode_style['fgcolor'] = 'red'
FZnode_style['size'] = 20
FZnode_style['hz_line_width'] = 5
FZnode_style['vt_line_width'] = 5
YZnode_style = NodeStyle()
YZnode_style['fgcolor'] = 'blue'
YZnode_style['size'] = 20
YZnode_style['hz_line_width'] = 5
YZnode_style['vt_line_width'] = 5
internal_node = NodeStyle()
internal_node['size'] = 0
internal_node['hz_line_width'] = 5
internal_node['vt_line_width'] = 5

for geneID, tree in pass_trees:
    for node in tree.traverse():
        if node.is_leaf():
            if node.name.startswith('NH_'):
                node.set_style(FZnode_style)
            elif node.name.startswith('XP_'):
                node.set_style(YZnode_style)
        else:
            node.set_style(internal_node)
    tree.render(os.path.join(outdir, '{}.png'.format(geneID)), tree_style=tree_style)
```

Supplementary Figure 1. The Kmer analysis curve generated by GenomeScope (related to Figure 1).

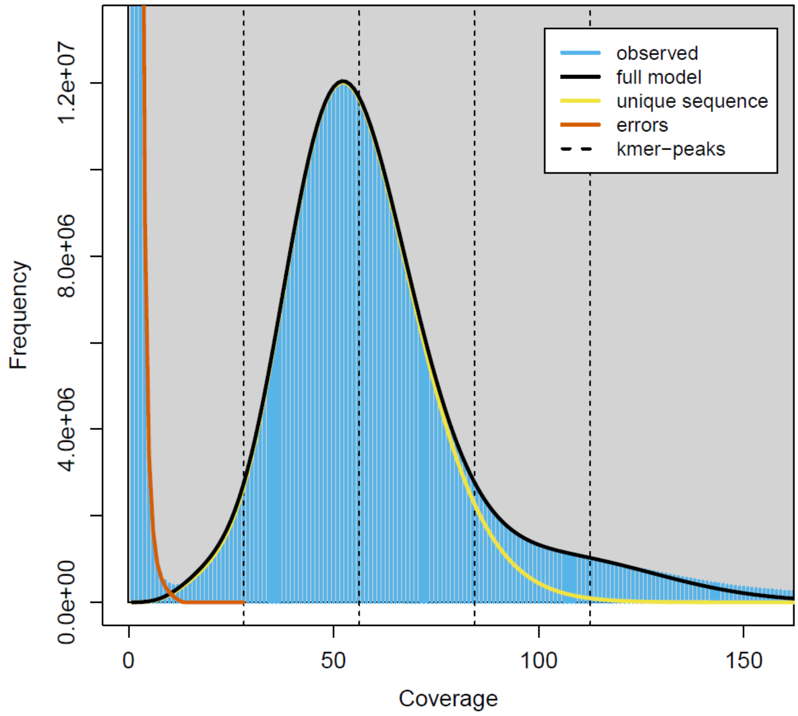

Supplementary Figure 2. The Hi-C assembly correlation heatmap (related to Figure 1).

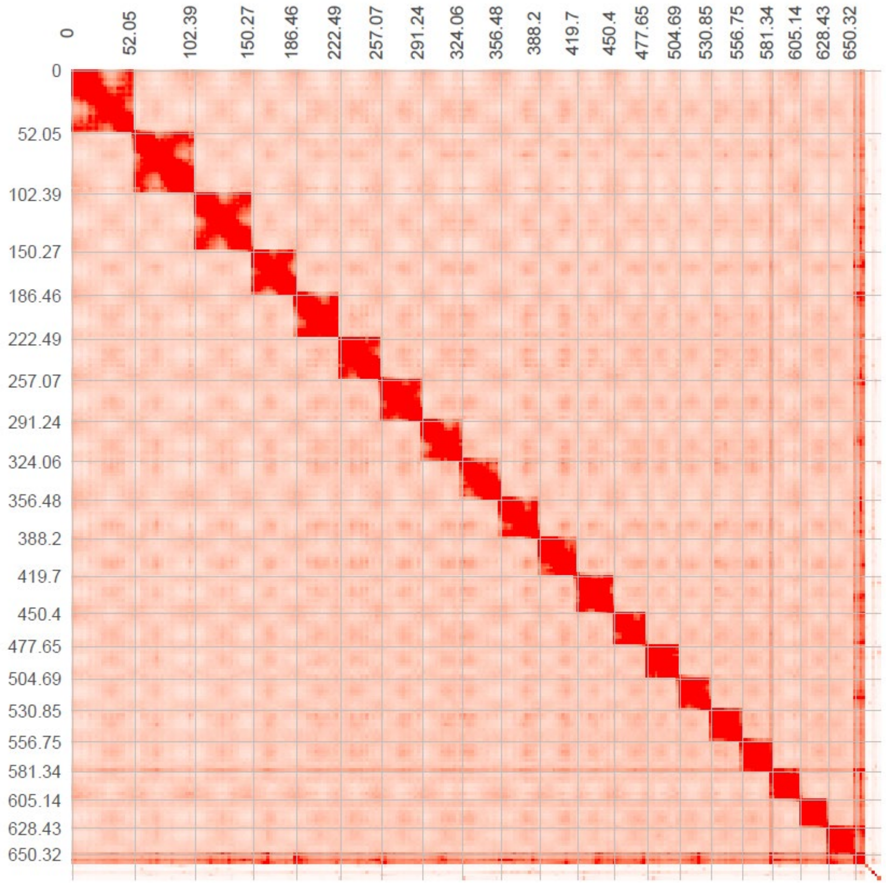

Supplementary Figure 3. The alignment spot plot of assembled and published mitochondrial genome of African arowana (related to Figure 1).

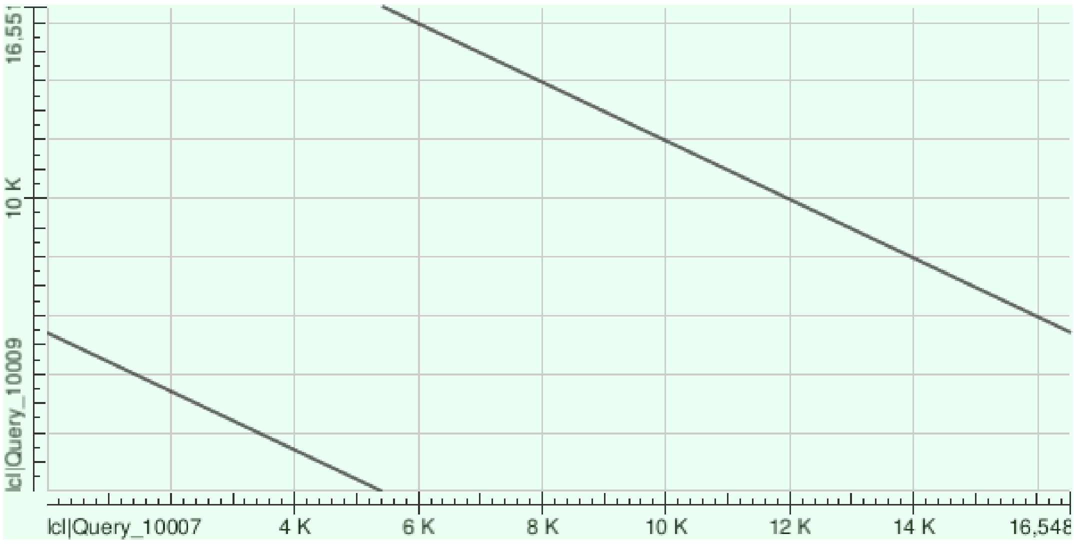

Supplementary Figure 4. The conserved regions genetic distance distribution (related to Figure 2).

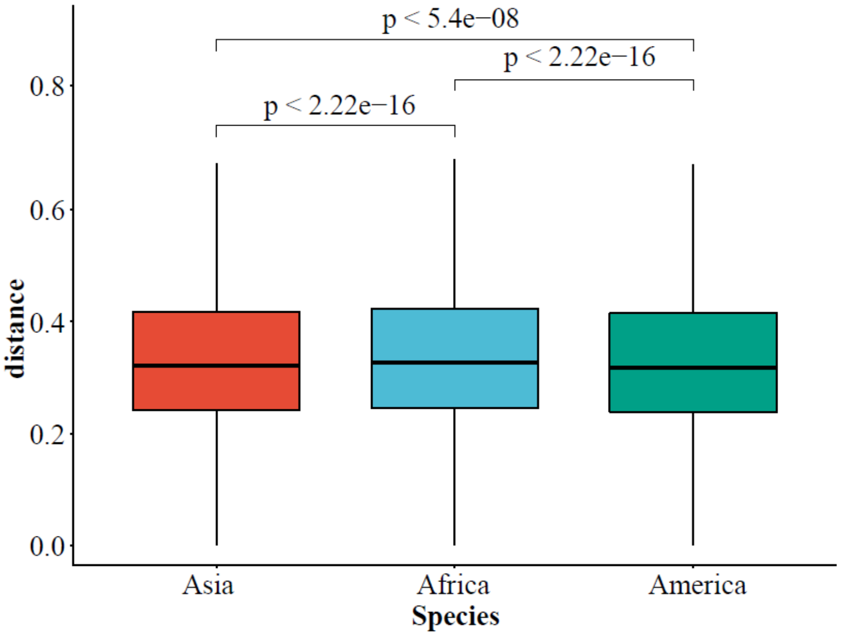

Supplementary Figure 5. The positional relationship of TEs and genes (related to Figure 3).

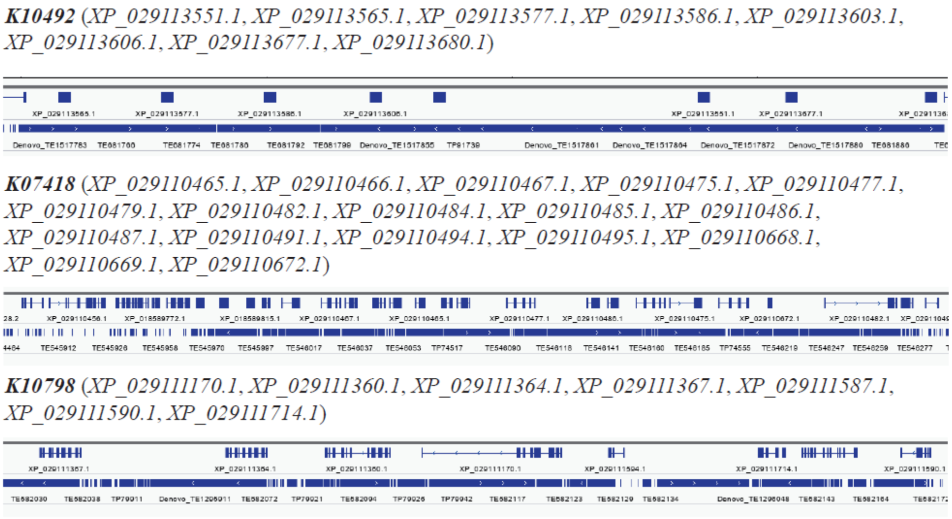

Supplementary Figure 6. Gene family expansion and contraction (related to Figure 4).

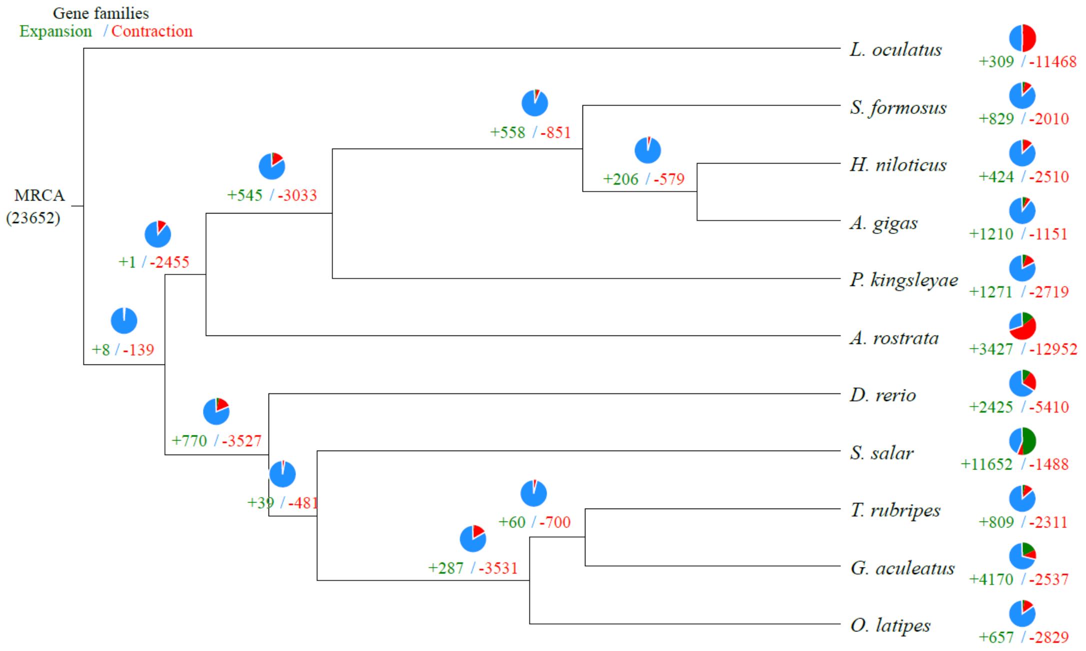

Supplementary Figure 7. 5S phylogenetic tree of three *Osteoglossidae* fishes (related to Figure 3).

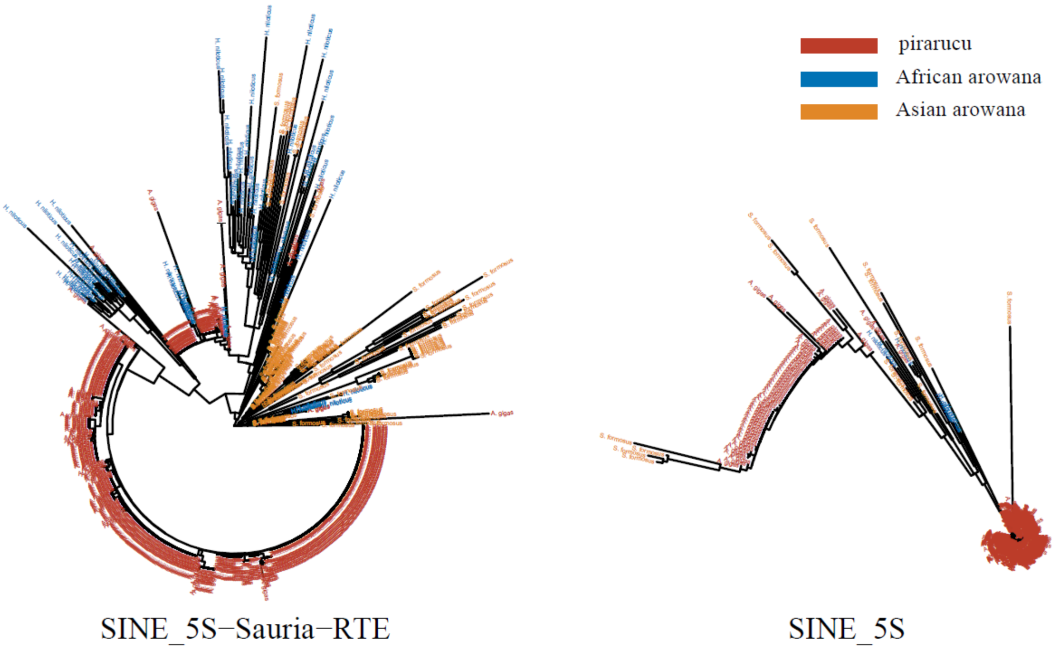

Supplementary table 1. Sequenced data statistics (related to Figure 1).

Category	Bases number(bp)	genome coverage	read length
**stLFR**	144,357,156,834	186.42	PE 100+140
**Nanopore**	10,172,388,541	13.14	Mean 20,695
**Hi-C**	21,234,895,000	27.42	PE 100+100

Supplementary table 2. Assemblies statistics (related to Figure 1).

#AssemblyName	#TotalScf	#ScfLen	#ScfN50	#ScfN90	#CtgNum	#CtgLen	#CtgN50	#CtgMax	#GC(%)
**stLFR**	4,244	669,728,546	9,615,753	2,219,485	20,530	663,211,256	75,203	520,346	43.04
**Gapcloser**	4,244	668,965,070	9,607,086	2,217,247	8,985	664,130,755	255,609	2,063,305	43.04
**Gapcloser_hic**	4,031	669,071,570	32,202,068	23,657,292	8,985	664,130,755	255,609	2,063,305	43.04
**TGS_gapcloser**	4,244	671,332,112	9,354,403	2,215,192	4,685	670,817,437	2,307,881	10,206,657	43.04
**TGS_gapcloser_hic**	3,995	671,456,612	32,427,226	23,803,277	4,685	670,817,437	2,307,881	10,206,657	43.04

Supplementary table 3. Chromosome statistics (related to Figure 1).

Chromosome	Length	Gene num	Gene num per Mb
chr1	51,871,654	1,789	34.49
chr2	49,976,607	1,704	34.10
chr3	47,507,736	1,693	35.64
chr4	36,097,016	1,096	30.36
chr5	35,841,748	1,402	39.12
chr6	34,505,377	1,317	38.17
chr7	34,149,782	1,158	33.91
chr8	32,770,312	1,234	37.66
chr9	32,202,068	1,476	45.84
chr10	31,542,738	1,291	40.93

chr11	31,366,365	1,152	36.73
chr12	30,527,933	1,199	39.28
chr13	27,111,882	1,156	42.64
chr14	27,037,882	963	35.62
chr15	25,963,407	952	36.67
chr16	25,831,899	937	36.27
chr17	24,583,205	798	32.46
chr18	23,657,292	707	29.89
chr19	23,251,436	783	33.68
chr20	21,895,334	666	30.42

Supplementary table 4. BUSCO evaluation statistics (related to Figure 1).

	Complete(C)	Complete and single-copy(S)	Complete and duplicated(D)	Fragmented(F)	Missing(M)	Total
**Genome**	2,522	2,303	219	33	31	2,586
**Gene set**	2,503	2,239	264	54	29	2,586

Supplementary table 5. Gene orthologous statistics in public database (related to Figure 1).

Values	Total	Nr-Annotated	Swissprot-Annotated	KEGG-Annotated	TrEMBL-Annotated	Overall
**Number**	24,146	21,553	20,915	19,504	21,597	21,621
**Percentage**	100%	89.26%	86.62%	80.78%	89.44%	89.54%

Supplementary table 6. Fossil records used for divergence time calibration (related to Figure 1).

Species pairs	Calibration time
Lepisosteus oculatus vs *Anguilla rostrata*	295-334 MYA
Anguilla rostrate vs *Heterotis niloticus*	244-295 MYA
Salmo salar vs *Heterotis niloticus*	231-287 MYA

Oryzias latipes vs *Danio rerio*	206–252 MYA

Supplementary table 7. Repeat annotation statistics (related to Figure 3).

		**Repbase TEs**		**De novo**		**Combined TEs**			
		**Length**	**percent**	**Length**	**percent**	**Length**	**percent**	**Length**	**percent**
H. niloticus	**DNA**	29,035,142	4.34	10,045,489	1.50	53,173,290	7.95	61,884,787	9.25
	**LINE**	17,099,871	2.56	14,416,154	2.15	31,437,295	4.70	37,093,168	5.54
	**SINE**	2,735,885	0.41	0	0.00	13,704,234	2.05	15,532,601	2.32
	**LTR**	4,909,599	0.73	2,355,009	0.35	18,023,858	2.69	20,631,561	3.08
	**Other**	43,744	0.01	0	0.00	0	0.00	43,744	0.01
	**Unknown**	0	0.00	0	0.00	17,467,985	2.61	17,467,985	2.61
	**Total**	48,975,430	7.32	26,801,920	4.01	119,546,525	17.87	125,391,143	18.74
S. formosus	**DNA**	42,415,841	5.41	175,473	0.02	61,270,356	7.81	87,560,384	11.16
	**LINE**	34,463,839	4.39	27,564,392	3.51	115,833,482	14.76	128,335,906	16.36
	**SINE**	10,836,721	1.38	0	0.00	5,937,128	0.76	16,323,535	2.08
	**LTR**	13,209,567	1.68	11,278,450	1.44	92,879,447	11.84	96,772,766	12.33
	**Other**	21,115	0.00	0	0.00	0	0.00	21,115	0.00
	**Unknown**	0	0.00	0	0.00	2,261,502	0.29	2,261,502	0.29
	**Total**	90,376,886	11.52	39,002,653	4.97	223,604,261	28.50	231,738,076	29.54
A. gigas	**DNA**	25,710,824	3.85	8,357,531	1.25	68,201,272	10.22	75,867,848	11.37
	**LINE**	7,978,164	1.20	5,907,459	0.89	16,704,546	2.50	22,111,177	3.31
	**SINE**	3,446,846	0.52	0	0.00	8,206,595	1.23	11,014,888	1.65
	**LTR**	6,016,996	0.90	3,889,739	0.58	26,970,615	4.04	30,740,524	4.61
	**Other**	16,768	0.00	129	0.00	0	0.00	16,897	0.00
	**Unknown**	0	0.00	0	0.00	10,605,989	1.59	10,605,989	1.59
	**Total**	37,937,812	5.68	18,144,719	2.72	113,468,148	17.00	121,187,526	18.16

Supplementary Table 8. Transposable elements covered genes related pathways (related to Figure 4).

	#Pathway	CovergeBigThan0.9	All-gene	Pvalue	Qvalue
A. gigas	NOD-like receptor signaling pathway	56	404	1.31E-33	1.93E-31
	Necroptosis	47	349	1.03E-27	7.56E-26
	Antigen processing and presentation	30	151	4.58E-23	2.25E-21
	Tight junction	39	509	1.42E-14	5.21E-13
	Salivary secretion	26	279	5.36E-12	1.58E-10
	Mitophagy - animal	17	148	1.11E-09	2.71E-08
	Endocytosis	32	603	3.50E-08	7.35E-07
	Longevity regulating pathway - multiple species	15	160	1.64E-07	3.02E-06
	Spliceosome	16	254	1.24E-05	1.86E-04
	Arginine and proline metabolism	9	81	1.27E-05	1.86E-04
	Estrogen signaling pathway	16	271	2.75E-05	3.68E-04
	PPAR signaling pathway	9	132	0.000561226	6.05E-03
	Protein processing in endoplasmic reticulum	15	319	0.00057866	6.05E-03
	Olfactory transduction	11	191	0.000599972	6.05E-03
	MAPK signaling pathway	25	693	0.000617763	6.05E-03
	Cardiac muscle contraction	10	164	0.000678535	6.23E-03
H. niloticus	NOD-like receptor signaling pathway	12	288	4.24E-10	3.18E-08
S.formosus	Olfactory transduction	124	277	7.43E-81	1.49E-78
	NOD-like receptor signaling pathway	117	429	5.45E-49	5.45E-47
	Phagosome	84	327	3.28E-33	2.19E-31
	Linoleic acid metabolism	31	61	4.48E-23	2.24E-21
	Ovarian steroidogenesis	34	113	1.84E-16	7.36E-15
	Arachidonic acid metabolism	30	103	2.88E-14	9.61E-13

	Salivary secretion	46	254	1.25E-12	3.57E-11
	Necroptosis	51	319	1.03E-11	2.57E-10
	Antigen processing and presentation	30	138	1.02E-10	2.28E-09
	Cytokine-cytokine receptor interaction	50	402	9.60E-08	1.92E-06
	Aminoacyl-tRNA biosynthesis	23	118	1.30E-07	2.37E-06
	Inflammatory mediator regulation of TRP channels	33	237	1.20E-06	2.01E-05
	alpha-Linolenic acid metabolism	12	42	2.01E-06	2.97E-05
	Apoptosis	38	300	2.09E-06	2.97E-05
	Neuroactive ligand-receptor interaction	62	600	2.23E-06	2.97E-05
	Serotonergic synapse	31	226	3.45E-06	4.31E-05
	TGF-beta signaling pathway	32	245	6.89E-06	8.10E-05
	Longevity regulating pathway - worm	25	170	8.76E-06	9.73E-05
	Gap junction	26	189	1.96E-05	2.06E-04
	Intestinal immune network for IgA production	18	109	3.24E-05	3.24E-04
	NF-kappa B signaling pathway	27	224	0.00014015	1.33E-03
	RIG-I-like receptor signaling pathway	19	146	0.000504316	4.58E-03
	Ether lipid metabolism	14	92	0.000562398	4.89E-03
	Mitophagy - animal	18	140	0.000808824	6.74E-03

Supplemental table 9. Unique gene families related pathways (related to Figure 4).

	#Pathway	unique_fam	All-gene	Pvalue	Qvalue
A. gigas	NOD-like receptor signaling pathway	84	404	2.44E-20	5.09E-18
	Necroptosis	75	349	3.72E-19	3.89E-17
	Phagosome	67	319	1.03E-16	7.19E-15
	Tight junction	88	509	6.07E-16	3.17E-14
	Cell adhesion molecules (CAMs)	80	477	7.20E-14	3.01E-12

	ECM-receptor interaction	46	238	1.30E-10	4.52E-09
	Mismatch repair	18	50	2.20E-09	5.77E-08
	Apoptosis	56	349	2.21E-09	5.77E-08
	Adherens junction	43	257	4.64E-08	1.08E-06
	Gap junction	38	249	2.84E-06	5.93E-05
	Focal adhesion	65	533	4.41E-06	8.39E-05
	Antigen processing and presentation	26	151	1.19E-05	2.07E-04
	Hematopoietic cell lineage	26	153	1.51E-05	2.43E-04
	Carbohydrate digestion and absorption	15	83	0.000469753	7.01E-03
H. niloticus	Ascorbate and aldarate metabolism	9	27	8.02E-12	1.37E-09
	Pentose and glucuronate interconversions	9	35	1.12E-10	9.57E-09
	Drug metabolism - cytochrome P450	9	44	1.03E-09	5.88E-08
	Retinol metabolism	10	67	3.08E-09	1.32E-07
	Steroid hormone biosynthesis	9	55	8.33E-09	2.85E-07
	Porphyrin and chlorophyll metabolism	8	46	3.48E-08	9.93E-07
	Drug metabolism - other enzymes	8	49	5.85E-08	1.25E-06
	Metabolism of xenobiotics by cytochrome P450	8	49	5.85E-08	1.25E-06
S. formosus	NOD-like receptor signaling pathway	67	429	1.04E-37	2.11E-35
	Salivary secretion	30	254	5.43E-14	5.54E-12
	Olfactory transduction	26	277	4.69E-10	3.19E-08
	Notch signaling pathway	13	125	3.50E-06	1.79E-04
	Oxidative phosphorylation	14	174	2.91E-05	1.19E-03
	Dorso-ventral axis formation	11	113	3.67E-05	1.25E-03
	Cell adhesion molecules (CAMs)	23	416	4.70E-05	1.37E-03
	PPAR signaling pathway	11	124	8.61E-05	2.20E-03
	Phenylalanine, tyrosine and tryptophan biosynthesis	4	14	0.000186839	4.24E-03

	Long-term potentiation	12	171	0.000382786	7.81E-03
	Phenylalanine metabolism	5	30	0.000435588	8.08E-03

Supplementary Table 10. The expanded gene families related pathways (related to Figure 4).

	#Pathway	Expanded gene number	All gene number	Pvalue	Qvalue
A.gigas	Cell adhesion molecules (CAMs)	156	477	1.12E-22	2.58E-20
	NOD-like receptor signaling pathway	130	404	1.92E-18	2.21E-16
	Olfactory transduction	69	191	4.40E-13	3.37E-11
	Salivary secretion	89	279	7.97E-13	4.58E-11
	Intestinal immune network for IgA production	43	107	2.24E-10	8.79E-09
	Antigen processing and presentation	54	151	2.29E-10	8.79E-09
	Tight junction	128	509	1.17E-09	3.85E-08
	Cardiac muscle contraction	54	164	6.92E-09	1.99E-07
	Necroptosis	92	349	2.32E-08	5.92E-07
	Phagosome	85	319	4.65E-08	1.07E-06
	Mismatch repair	23	50	1.84E-07	3.86E-06
	Insulin secretion	61	213	2.47E-07	4.73E-06
	Gap junction	67	249	8.08E-07	1.43E-05
	Adrenergic signaling in cardiomyocytes	95	392	9.43E-07	1.55E-05
	Carbohydrate digestion and absorption	30	83	1.68E-06	2.58E-05
	Protein digestion and absorption	74	290	1.98E-06	2.85E-05
	Estrogen signaling pathway	66	271	3.43E-05	4.64E-04
	Notch signaling pathway	40	146	8.04E-05	1.03E-03
	Proximal tubule bicarbonate reclamation	19	55	2.58E-04	3.13E-03
	Inflammatory mediator regulation of TRP channels	57	243	3.23E-04	3.71E-03
	Phototransduction - fly	26	91	6.72E-04	7.36E-03

H.niloticus	Drug metabolism - cytochrome P450	14	44	3.16E-08	6.13E-06
	Ascorbate and aldarate metabolism	10	27	6.03E-07	4.59E-05
	Progesterone-mediated oocyte maturation	26	167	7.10E-07	4.59E-05
	Drug metabolism - other enzymes	13	49	1.03E-06	5.02E-05
	Arginine and proline metabolism	16	81	4.33E-06	1.68E-04
	Retinol metabolism	14	67	8.61E-06	2.78E-04
	Retrograde endocannabinoid signaling	29	241	3.12E-05	8.64E-04
	Metabolism of xenobiotics by cytochrome P450	11	49	3.90E-05	9.45E-04
	Regulation of lipolysis in adipocytes	19	134	8.09E-05	1.74E-03
	Necroptosis	29	259	1.15E-04	2.23E-03
	Adrenergic signaling in cardiomyocytes	34	336	2.26E-04	3.99E-03
	Selenocompound metabolism	7	26	3.06E-04	4.38E-03
	Gap junction	23	197	3.09E-04	4.38E-03
	NOD-like receptor signaling pathway	30	288	3.16E-04	4.38E-03
	Pentose and glucuronate interconversions	8	35	3.89E-04	5.03E-03
	Adipocytokine signaling pathway	18	140	4.26E-04	5.16E-03
	Glucagon signaling pathway	22	193	5.76E-04	6.26E-03
	Longevity regulating pathway - multiple species	16	120	5.81E-04	6.26E-03
	Gastric acid secretion	21	184	7.48E-04	7.64E-03
	Bile secretion	17	136	8.40E-04	8.15E-03
	MAPK signaling pathway - fly	22	200	9.31E-04	8.60E-03
	Cholinergic synapse	25	241	1.02E-03	9.01E-03
	Protein processing in endoplasmic reticulum	28	284	1.16E-03	9.80E-03
S.formosus	Olfactory transduction	174	277	5.53E-82	1.27E-79
	Phagosome	151	327	2.53E-47	2.90E-45
	NOD-like receptor signaling pathway	159	429	2.73E-35	2.09E-33

Cell adhesion molecules (CAMs)	140	416	2.88E-26	1.66E-24
Linoleic acid metabolism	41	61	6.95E-22	3.20E-20
Intestinal immune network for IgA production	56	109	3.84E-21	1.47E-19
Antigen processing and presentation	60	138	5.59E-18	1.84E-16
Salivary secretion	80	254	8.82E-14	2.54E-12
Hematopoietic cell lineage	60	174	1.51E-12	3.85E-11
Necroptosis	90	319	3.00E-12	6.91E-11
Arachidonic acid metabolism	42	103	6.80E-12	1.42E-10
Ovarian steroidogenesis	44	113	1.40E-11	2.67E-10
Complement and coagulation cascades	59	186	1.12E-10	1.98E-09
Cytokine-cytokine receptor interaction	99	402	1.15E-09	1.90E-08
Inflammatory mediator regulation of TRP channels	67	237	1.60E-09	2.45E-08
Phototransduction	32	82	7.71E-09	1.11E-07
TGF-beta signaling pathway	66	245	1.73E-08	2.34E-07
alpha-Linolenic acid metabolism	18	42	2.96E-06	3.77E-05
Vascular smooth muscle contraction	63	263	3.11E-06	3.77E-05
Gastric acid secretion	48	183	3.30E-06	3.80E-05
Aminoacyl-tRNA biosynthesis	35	118	3.67E-06	4.02E-05
Phototransduction - fly	27	81	4.18E-06	4.37E-05
NF-kappa B signaling pathway	55	224	5.89E-06	5.89E-05
Protein digestion and absorption	57	239	1.03E-05	9.89E-05
Mineral absorption	23	67	1.22E-05	1.12E-04
GnRH signaling pathway	52	214	1.43E-05	1.26E-04
Cellular senescence	72	338	4.91E-05	4.18E-04
Gap junction	45	189	8.75E-05	7.18E-04
RIG-I-like receptor signaling pathway	37	146	9.20E-05	7.30E-04

Estrogen signaling pathway	52	232	1.39E-04	1.06E-03
Long-term potentiation	40	171	3.10E-04	2.30E-03
Apoptosis	62	300	3.74E-04	2.69E-03
Oxytocin signaling pathway	69	347	5.70E-04	3.97E-03
Bile secretion	33	140	8.64E-04	5.84E-03
Ether lipid metabolism	24	92	9.52E-04	6.26E-03
Apelin signaling pathway	59	295	1.19E-03	7.41E-03
Proximal tubule bicarbonate reclamation	15	48	1.19E-03	7.41E-03
Neurotrophin signaling pathway	55	274	1.56E-03	9.21E-03
Serotonergic synapse	47	226	1.56E-03	9.21E-03

Supplementary table 11. The contracted gene families of African arowana related pathways (related to Figure 4).

#Pathway	Bonytongue.Henil.contracted (757)	All-gene (19504)	Pvalue	Qvalue
**Olfactory transduction**	30	150	1.04E-13	2E-11
**Tight junction**	33	372	9.48E-06	0.0008
**Cellular senescence**	27	278	1.22E-05	0.0008
**TGF-beta signaling pathway**	21	200	3.54E-05	0.0018
**Intestinal immune network for IgA production**	11	66	4.28E-05	0.0018
**Cell adhesion molecules (CAMs)**	27	305	6.23E-05	0.0022
**Vitamin digestion and absorption**	9	48	8.21E-05	0.0024
**Cytokine-cytokine receptor interaction**	27	313	9.66E-05	0.0025
**Signaling pathways regulating pluripotency of stem cells**	25	297	0.00025	0.0059

Supplementary table 12. Taste receptors statistics (related to Figure 4).

	A. gigas	*H. niloticus*	*S. formosus*

**K04624_TIR1 (Umami)**	4	4	4
**K04626_TIR3 (Sweet&Umami)**	3	2	3
**K08474_TAS2R (Bitter)**	3	4	2
**K04990_PKD2L1 (Sour)**	1	1	1
**K04824_SCNN1A (Salty)**	1	1	1

Supplementary table 13. Odorant receptors statistics (related to Figure 4).

	A. gigas	*H. niloticus*	*S. formosus*
**K04257_OR**	70	45	160
**K04614_V1R**	14	15	14
**K04613_V2R**	2	2	1

Reference

Benson, G. (1999). Tandem repeats finder: a program to analyze DNA sequences. Nucleic Acids Res *27*, 573-580.

Birney, E., Clamp, M., and Durbin, R. (2004). GeneWise and Genomewise. Genome Res *14*, 988-995.

Burge, C., and Karlin, S. (1997). Prediction of complete gene structures in human genomic DNA. J Mol Biol *268*, 78-94.

De Bie, T., Cristianini, N., Demuth, J.P., and Hahn, M.W. (2006). CAFE: a computational tool for the study of gene family evolution. Bioinformatics *22*, 1269-1271.

Dudchenko, O., Batra, S.S., Omer, A.D., Nyquist, S.K., Hoeger, M., Durand, N.C., Shamim, M.S., Machol, I., Lander, E.S., Aiden, A.P.*, et al.* (2017). De novo assembly of the Aedes aegypti genome using Hi-C yields chromosome-length scaffolds. Science *356*, 92-95.

Durand, N.C., Robinson, J.T., Shamim, M.S., Machol, I., Mesirov, J.P., Lander, E.S., and Aiden, E.L. (2016a). Juicebox Provides a Visualization System for Hi-C Contact Maps with Unlimited Zoom. Cell Syst *3*, 99-101.

Durand, N.C., Shamim, M.S., Machol, I., Rao, S.S., Huntley, M.H., Lander, E.S., and Aiden, E.L. (2016b). Juicer Provides a One-Click System for Analyzing Loop-Resolution Hi-C Experiments. Cell Syst *3*, 95-98.

Edgar, R.C. (2004). MUSCLE: multiple sequence alignment with high accuracy and high throughput. Nucleic Acids Res *32*, 1792-1797.

Elsik, C.G., Mackey, A.J., Reese, J.T., Milshina, N.V., Roos, D.S., and Weinstock, G.M. (2007). Creating a honey bee consensus gene set. Genome Biol *8*, R13. doi: 10.1186/gb-2007-8-1-r13

Huerta-Cepas, J., Serra, F., and Bork, P. (2016). ETE 3: Reconstruction, Analysis, and Visualization of Phylogenomic Data. Mol Biol Evol *33*, 1635-1638.

Katoh, K., and Standley, D.M. (2013). MAFFT multiple sequence alignment software version 7: improvements in performance and usability. Mol Biol Evol *30*, 772-780.

Kim, D., Paggi, J.M., Park, C., Bennett, C., and Salzberg, S.L. (2019). Graph-based genome alignment and genotyping with HISAT2 and HISAT-genotype. Nat Biotechnol *37*, 907-915.

Kumar, S., Stecher, G., Suleski, M., and Hedges, S.B. (2017). TimeTree: A Resource for Timelines, Timetrees, and Divergence Times. Mol Biol Evol *34*, 1812-1819.

Letunic, I., and Bork, P. (2019). Interactive Tree Of Life (iTOL) v4: recent updates and new developments. Nucleic Acids Res *47*, W256-W259.

Luo, R., Liu, B., Xie, Y., Li, Z., Huang, W., Yuan, J., He, G., Chen, Y., Pan, Q., Liu, Y.*, et al.* (2012). SOAPdenovo2: an empirically improved memory-efficient short-read de novo assembler. Gigascience *1*, 18. doi: 10.1186/2047-217X-1-18

Majoros, W.H., Pertea, M., and Salzberg, S.L. (2004). TigrScan and GlimmerHMM: two open source ab initio eukaryotic gene-finders. Bioinformatics *20*, 2878-2879.

Marcais, G., and Kingsford, C. (2011). A fast, lock-free approach for efficient parallel counting of occurrences of k-mers. Bioinformatics *27*, 764-770.

Price, M.N., Dehal, P.S., and Arkin, A.P. (2010). FastTree 2--approximately maximum-likelihood trees for large alignments. PLoS One *5*, e9490. doi: 10.1371/journal.pone.0009490

Ronquist, F., Teslenko, M., van der Mark, P., Ayres, D.L., Darling, A., Hohna, S., Larget, B., Liu, L., Suchard, M.A., and Huelsenbeck, J.P. (2012). MrBayes 3.2: efficient Bayesian phylogenetic inference and model choice across a large model space. Syst Biol *61*, 539-542.

Servant, N., Varoquaux, N., Lajoie, B.R., Viara, E., Chen, C.J., Vert, J.P., Heard, E., Dekker, J., and Barillot, E. (2015). HiC-Pro: an optimized and flexible pipeline for Hi-C data processing. Genome Biol *16*, 259. doi: 10.1186/s13059-015-0831-x

Stanke, M., Keller, O., Gunduz, I., Hayes, A., Waack, S., and Morgenstern, B. (2006). AUGUSTUS: ab initio prediction of alternative transcripts. Nucleic Acids Res *34*, W435-439.

Vurture, G.W., Sedlazeck, F.J., Nattestad, M., Underwood, C.J., Fang, H., Gurtowski, J., and Schatz, M.C. (2017). GenomeScope: fast reference-free genome profiling from short reads. Bioinformatics *33*, 2202-2204.

Wang, O., Chin, R., Cheng, X., Wu, M.K.Y., Mao, Q., Tang, J., Sun, Y., Anderson, E., Lam, H.K., Chen, D*., et al.* (2019). Efficient and unique cobarcoding of second-generation sequencing reads from long DNA molecules enabling cost-effective and accurate sequencing, haplotyping, and de novo assembly. Genome Res *29*, 798-808.

Waterhouse, R.M., Seppey, M., Simao, F.A., Manni, M., Ioannidis, P., Klioutchnikov, G., Kriventseva, E.V., and Zdobnov, E.M. (2018). BUSCO Applications from Quality Assessments to Gene Prediction and Phylogenomics. Mol Biol Evol *35*, 543-548.

Weisenfeld, N.I., Kumar, V., Shah, P., Church, D.M., and Jaffe, D.B. (2017). Direct determination of diploid genome sequences. Genome Res *27*, 757-767.

Xu, M., Guo, L., Gu, S., Wang, O., Zhang, R., Fan, G., Xu, X., Deng, L., and Liu, X. (2019). TGS-GapCloser: fast and accurately passing through the Bermuda in large genome using error-prone third-generation long reads. bioRxiv. doi: 10.1101/831248

Xu, Z., and Wang, H. (2007). LTR_FINDER: an efficient tool for the prediction of full-length LTR retrotransposons. Nucleic Acids Res *35*, W265-268.

Yang, Z. (2007). PAML 4: phylogenetic analysis by maximum likelihood. Mol Biol Evol *24*, 1586-1591.

Yu, G., Smith, D.K., Zhu, H., Guan, Y., Lam, T.T.-Y., and McInerny, G. (2017). ggtree: anrpackage for visualization and annotation of phylogenetic trees with their covariates and other associated data. Methods in Ecology and Evolution *8*, 28-36.

Zhang, Z., Carriero, N., Zheng, D., Karro, J., Harrison, P.M., and Gerstein, M. (2006). PseudoPipe: an automated pseudogene identification pipeline. Bioinformatics *22*, 1437-1439.

Zwaenepoel, A., and Van de Peer, Y. (2019). wgd-simple command line tools for the analysis of ancient whole-genome duplications. Bioinformatics *35*, 2153-2155.
